# A new machine learning method for cancer mutation analysis

**DOI:** 10.1371/journal.pcbi.1010332

**Published:** 2022-10-17

**Authors:** Mahnaz Habibi, Golnaz Taheri

**Affiliations:** 1 Department of Mathematics, Qazvin Branch, Islamic Azad University, Qazvin, Iran; 2 Department of Electrical Engineering and Computer Science, KTH Royal Institute of Technology, Stockholm, Sweden; 3 Science for Life Laboratory, Stockholm, Sweden; Queen’s University, CANADA

## Abstract

It is complicated to identify cancer-causing mutations. The recurrence of a mutation in patients remains one of the most reliable features of mutation driver status. However, some mutations are more likely to happen than others for various reasons. Different sequencing analysis has revealed that cancer driver genes operate across complex pathways and networks, with mutations often arising in a mutually exclusive pattern. Genes with low-frequency mutations are understudied as cancer-related genes, especially in the context of networks. Here we propose a machine learning method to study the functionality of mutually exclusive genes in the networks derived from mutation associations, gene-gene interactions, and graph clustering. These networks have indicated critical biological components in the essential pathways, especially those mutated at low frequency. Studying the network and not just the impact of a single gene significantly increases the statistical power of clinical analysis. The proposed method identified important driver genes with different frequencies. We studied the function and the associated pathways in which the candidate driver genes participate. By introducing lower-frequency genes, we recognized less studied cancer-related pathways. We also proposed a novel clustering method to specify driver modules. We evaluated each driver module with different criteria, including the terms of biological processes and the number of simultaneous mutations in each cancer. Materials and implementations are available at: https://github.com/MahnazHabibi/MutationAnalysis.

## Author summary

It can be challenging to find mutations that cause cancer. One of the most trustworthy characteristics for identifying cancer-causing mutations is the recurrence of a mutation in patients. However, some uncommon and low-frequency mutations should also be explored as cancer-related mutations, particularly in the setting of networks. In this study, we suggested a unique approach to discover prospective driver genes and investigate the functionality of mutually exclusive genes in networks formed from mutation connections and gene-gene interactions. These networks have identified critical biological elements in the vital pathways, notably in those that experience infrequent mutations. In the first step, we established six enlightening topological features for each gene acting as a network node. For each gene, we computed the score for our predefined features. Then, we suggested the high-scoring genes with significant connections to cancer as potential targets for further research. In the second step, we constructed a network based on the relationships between the high-score genes to find the cancer-related modules. We used what we had learned in the first step about how the high-score potential driver genes interact physically, biologically, and in terms of how they work to build this network.

## 1 Introduction

The driving forces behind cancer are gene, nucleotide, and cellular structure changes. Somatic cells can acquire mutations one or two orders of magnitude more quickly than germline cells, making them more susceptible to different types of cancer [1]. The vast majority of these mutations, called passenger, have little effect on cell proliferation compared to a few driver mutations that give cells a selective advantage [2]. Mutations can activate or deactivate proteins, and they can change a wide range of cellular processes for different patients and types of cancer. This results in high intra- and inter-tumor heterogeneity in biochemistry and histology, which may explain why some cancers are resistant to treatment and make it more challenging to identify the events that cause cancer [3–5].

The study of cancer genomes has been completely changed by next-generation sequencing technology, which allows us to analyze millions of cancer genomes in-depth and identify somatic mutations. The Cancer Genome Atlas (TCGA), a publicly funded genomics project, contains a collection of mutation profiles from thousands of patients for more than 30 different types of cancer [6]. The recent mutation perspective demonstrates the importance of specifying genes and their associated networks to detect the cancer driver genes [6]. Finding significantly mutated genes with high recurrent mutations can help us better predict the course of cancer development and progression. These cancer-causing driver genes are difficult to track down, and many of the mutations have not been detected using existing methods datasets. Methodical studies have shown multiple new genes and classes of cancer genes, respectively [7]. They have also demonstrated that despite some cancer genes being mutated with high frequencies, most cancer genes in most patients arise with intermediate or low frequencies (2–20%) [7]. Therefore, a complete record of mutations in this frequency class will be essential for identifying dysregulated pathways and effective targets for therapeutic interference [7]. Nevertheless, current studies present significant gaps in our understanding of cancer genes with intermediate frequency. For example, a study of 183 lung adenocarcinomas discovered that 15% of patients missed even a single mutation influencing any of the 10 known hallmarks of cancer, and 38% of patients had 3 or even fewer such mutations [8]. As a result, we cannot capture a complete expression profile of all genes and subsets of genes that drive the evolution and progression of cancer. Cancer genes tend to alter considerably in a limited number of pathways, especially in pathways related to survival, cell division, differentiation, and genomic preservation. Therefore, it is necessary to determine the pathway-level importance of genes, even those genes mutated at low frequencies [9].

Since mutually exclusive couples of genes usually share similar pathways, one strategy for detecting these drivers is to explore the mutual exclusivity of changed genes. However, we know that mutated genes seldom coexist in the same tumor, while only one gene in a pathway is typically found to have a driver mutation in each patient [10]. This situation may occur due to cancer pathways’ functional redundancy or synthetic lethality. Typical examples of mutually exclusive driver mutations contain EGFR and KRAS mutations in lung cancer [11] and TP53 and MDM2 mutations in glioblastoma [6]. Based on this explanation, finding mutual exclusivity modules in cancer needs to find important and more relevant genes, find the correlation between them, and analyze them. Then this analysis needs statistical tests to identify network modules demonstrating patterns of mutually exclusive genetic changes across multiple patients [12]. As a new method, Mutex uses a large pathway model of human signaling processes to explore groups of mutually exclusively changed genes that share a joint downstream event [13].

The main disadvantage of the current methods is that they need comprehensive filtering of mutation data, which are restricted to the most significantly mutated genes and concentrate on predefined network modules [14]. The mutual exclusivity signal may be biased towards recognizing gene sets where most of the coverage comes just from highly mutated genes [15, 16]. Although cancer-related genes have been shown to be involved in numerous pathways, few methods determine the important gene sets where a gene has various mutually exclusive correlations with other genes in diverse pathways at different mutation frequencies. Recently, different methods have been proposed to identify driver genes [17–23]. Some of these methods, such as WITER [17] and driverMAPS [18] are based on the driver genes mutation frequency. These methods are based on the idea that the mutation frequency in driver genes is higher than the background mutation frequency. Some other methods are network-based identification of promoter genes such as HotNet2 [19], NetSig [20], DNmax [21], nCOP [22] and MaxMIF [23]. In these methods, pathways, networks, and mutation frequencies are studied. Some of these network-based methods could identify the number of low-frequency mutated genes.

We proposed a novel two-step method to identify candidate driver gene sets with mutually exclusive mutations to more comprehensively find the mutually exclusive mutation pattern. In the first step, the proposed unsupervised machine learning method detects candidate driver genes from TCGA [6], which includes a mutation collection with low and high-frequency occurrence from thousands of patients for more than 30 different cancer types. For this purpose, we constructed a biological network corresponding to important cancer-related genes. Then, we defined six informative topological features for each gene as a node in the network. We calculated the score for our predefined features for each gene. Afterward, we introduced the high-score genes with meaningful relationships to cancer as candidates for more investigation. In the second step, we presented a network based on the relationship between genes to identify the cancer-related modules. We used the information on physical, biological, and functional interaction between the high-score candidate driver genes obtained in the first step to construct this network.

## 2 Materials and methods

**Fig 1:**
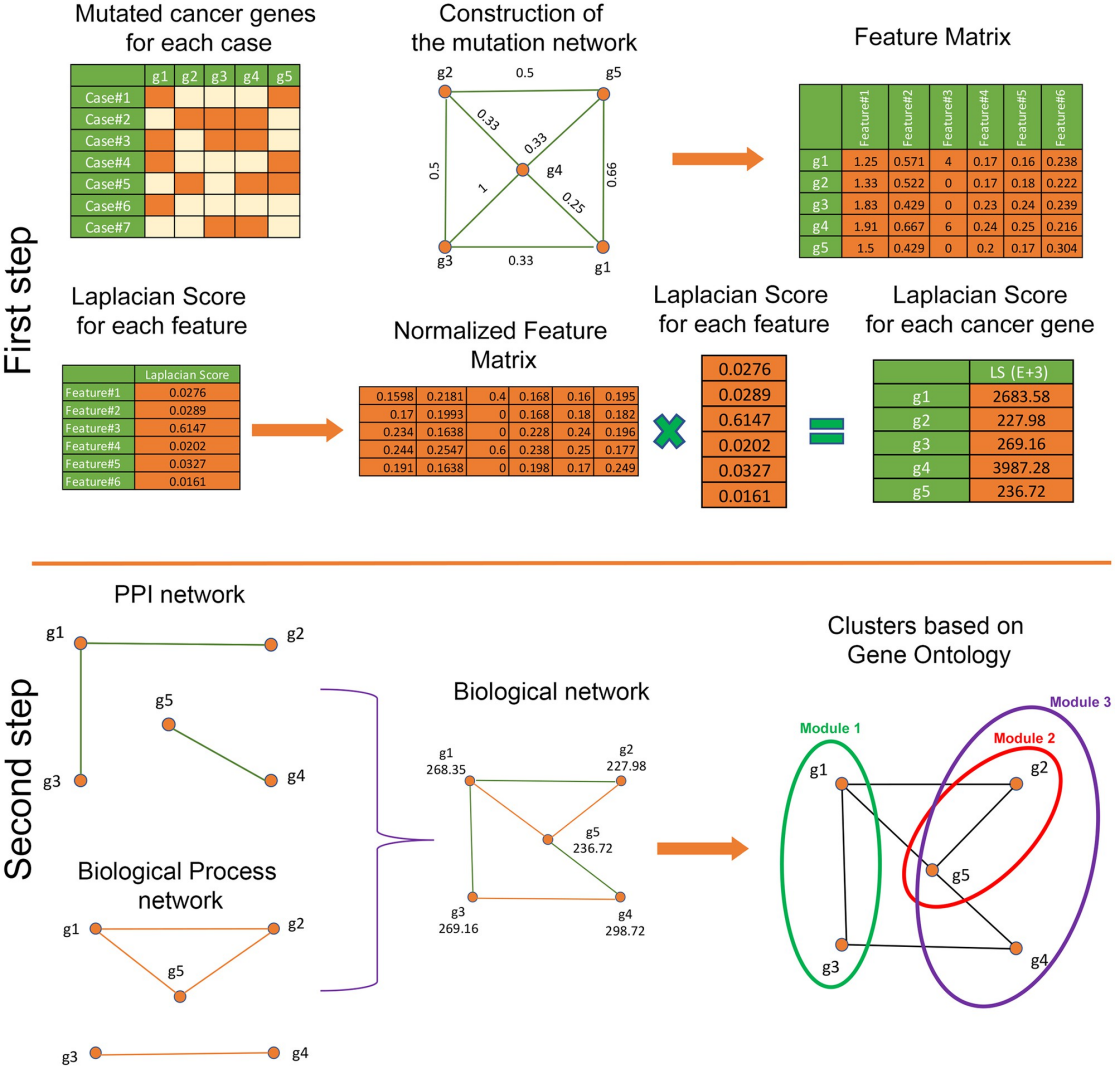
In the first step, we used the information of different patients with various types of cancer and their associated mutated genes to create the weighted network of mutated genes (*G*). Then, six informative topological features are calculated for each gene as a node of *G*. We used an unsupervised learning method to calculate the appropriate scores for each feature. Finally, our method selects a set of genes with higher scores as a set of mutated genes that contain valuable information. In the second step, we constructed another network based on the gene relationship to identify the cancer-related modules (G). To build this network, we used the information on biological interactions between the high-score genes obtained in the first step. Then, we used a heuristic *MG* method to find cancer driver modules in the constructed weighted network. The weight of each module is calculated based on the average weight of the nodes of that module.

In this section, we present a new two-step method for identifying driver genes and modules in different types of cancer. In the first step, we proposed an unsupervised machine learning method to recognize a set of candidate driver mutated genes associated with different types of cancer. In this step, we used the information of different patients (cases) with various types of cancer and their associated mutated genes to create a weighted network of mutated genes. Then six informative topological features are calculated for each gene as a node of the constructed network. We generated a feature matrix for the set of candidate mutated genes *X* = [*x*_*ij*_]_*m*×*n*_ that each *x*_*ij*_ component represents the *j*-th feature for the *i*-th gene. Then we employed an unsupervised learning method to calculate the appropriate scores for each of the predefined features. Finally, our proposed method selects a set of genes with higher scores as a set of mutated genes that contain valuable information. In the second step, we constructed a network based on the relationship between genes to identify the cancer-related driver modules. We used the information on biological and functional interactions between the high-score genes obtained in the first step to build this network. Then, we used a heuristic method to find cancer driver modules in the constructed weighted network. The weight of each driver module is calculated based on the average weight of the nodes of that module. The set of driver modules with higher weights is identified as the cancer-related modules containing important information. The general workflow of the proposed method is illustrated in Fig 1.

### 2.1 Datasets

Identifying associated driver genes with different cancer types plays a significant role in determining mutated driver modules. Therefore, the starting point is identifying appropriate datasets to extract complete information about the somatic mutation, corresponding protein-protein interaction (PPI), and biological process information. A representative set of tumors and mutations were gathered from TCGA, on March 2022 [6]. We downloaded the information on the primary site and mutations for 12,792 cases. This dataset contains 576 mutated cancer genes and 15 major primary sites. Since there is no comprehensive standard benchmark for collecting a set of driver genes, comparing driver gene detection methods is a significant challenge. In this work, we selected six independent data sets as benchmark sets. The first dataset contains 576 genes from the Cancer Gene Census (CGC; Tier 1; January 2019) [24]. This set, which we indicated by “CGC”, includes genes and mutations with oncogenic and cancer-related activity [24]. The second dataset contains a subset of 118 genes, which we demonstrated with “CGCpointMut”. This subset comprises genes participating in carcinogenesis through point mutations [24]. The third dataset, which we showed with the “Rule” contains 124 cancer genes that are included in oncogenes and tumor suppressor genes based on specific mutation patterns [9]. The fourth set contains 288 driver genes with high confidence predicted by at least two frequency-based methods. This gene set is denoted by the “HCD” [25]. The fifth set, CTAT (combined tool adjusted total), contains 297 driver genes [26]. The sixth set contains 232 driver genes, which are collected from the combination of seven driver gene sets (including five driver gene sets and two other gene sets). We denoted this set by the “ShiBench” [27].

We used the PPI network from Habibi et al. (2021) [28]. This dataset contains the physical interactions between proteins that are collected from the Biological General Repository for Interaction Datasets (BioGRID) [29], Agile Protein Interactomes Data analyzer (APID) [30], Homologous interactions (Hint) [31], Human Integrated Protein-Protein Interaction reference (HIPPIE) [32] and Huri [33]. All of the proteins in this dataset are mapped to a universal protein resource (UniProt) ID [34]. This interactome contains 20,040 proteins and 304,730 interactions. We also used the informative biological processes related to each mutated gene that is gathered from the Gene Ontology (GO) [35], to identify functional interactions between mutated cancer genes.

### 2.2 Construction of the mutation network

We introduced a mutation network based on 576 mutated cancer genes in this work. Suppose that *V* = {*g*_1_, …, *g*_*n*_} indicates the set of mutated cancer genes. Also, suppose that $C(g_{i})$ is the set of cases that contain a given mutation gene (*g*_*i*_). A weighted mutation network *G* = < *V*, *E*, *ω* > was constructed by connecting two genes *g*_*i*_ and *g*_*j*_ if and only if $C\left(g_{i}\right)\cap C\left(g_{j}\right)\neq \emptyset$. The weight of edge *g*_*i*_*g*_*j*_ ∈ *E* which is denoted by *ω*(*g*_*i*_*g*_*j*_), is defined as follows:

$$\begin{array}{c} \omega \left(g_{i}g_{j}\right)=\frac{|C\left(g_{i}\right)\cap C\left(g_{j}\right)|}{min\{|C\left(g_{i}\right)|,|C\left(g_{j}\right)|\}}. \end{array}$$

A path between *g*_*i*_ and *g*_*j*_ is determined as a sequence of distinct nodes such that an edge of *G* connects two consequent nodes. The weight of a path equals the sum of the weights of edges in this path. The shortest path from node *g*_*i*_ to node *g*_*j*_ is a path between two nodes with minimum weight. The weight of the shortest path between two nodes *g*_*i*_ and *g*_*j*_ is denoted by *d*_*w*_(*g*_*i*_, *g*_*j*_).

#### 2.2.1 Informative topological features for mutation network

We defined the following informative topological features for each node of the weighed mutated network.

- **Weight**: The Weight of node *g*_*i*_ on weighted graph *G* = < *V*, *E*, *ω* > as follows:
  $$\begin{array}{c} \omega \left(g_{i}\right)=\sum_{g_{j}\in V}\omega (g_{i}g_{j}). \end{array}$$ (1)
- **Closeness**: The Closeness centrality measure is defined for each node, *g*_*i*_, as follows:
  $$\begin{array}{c} C\left(g_{i}\right)=\frac{|V|-1}{\sum_{g_{j}\in V}d_{w}\left(g_{i},g_{j}\right)}. \end{array}$$ (2)
- **Betweenness**: The Betweenness centrality measure is defined of each node *g*_*i*_ on network *G* as follows:
  $$\begin{array}{c} B\left(g_{i}\right)=\sum_{g_{j}g_{k}\in V}\frac{\delta_{g_{j}g_{k}}\left(g_{i}\right)}{\delta_{g_{j}g_{k}}}, \end{array}$$ (3)
  where $\delta_{g_{j}g_{k}}$ denoted the weights of shortest paths between two nodes *g*_*i*_ and *g*_*k*_ and $\delta_{g_{j}g_{k}}\left(g_{i}\right)$ is indicated the weighs of shortest paths between two nodes *g*_*i*_ and *g*_*k*_ pass through node *g*_*i*_ [36].
- **PageRank**: The score for each node *g*_*i*_ in the network is calculated based on all the scores assigned to all nodes *g*_*j*_, which are connected iteratively as follows:
  $$\begin{array}{c} PR\left(g_{i}\right)=\left(1-d\right)+d*\left[\sum_{g_{i}\neq g_{j}}\frac{\omega (g_{i}g_{j})}{\sum_{g_{j}\neq g_{k}}\omega \left(g_{j}g_{k}\right)}PR\left(g_{j}\right)\right], \end{array}$$ (4)
  where *d* is a parameter between 0 and 1. In this work, we set the value of 0.85 for *d* [37].
- **Eigenvector**: The Eigenvector centrality measure is defined as the amount of influence for a node *g*_*i*_ in the network as follows:
  $$\begin{array}{c} EV\left(g_{i}\right)=\frac{1}{\lambda }\sum_{g_{j}\in V}\omega \left(g_{i}g_{j}\right)EV\left(g_{j}\right), \end{array}$$ (5)
  where λ is the maximum eigenvalue of the weighted adjacency matrix *A*_*w*_ = [*w*(*g*_*i*_, *g*_*j*_)]. The weighted adjacency matrix *A*_*w*_ is a weighted version of the adjacency matrix which contains the weight of each edge instead of 1 [38].
- **Entropy**: Suppose that *ω*(*g*_*i*_) is the weight of node *g*_*i*_ on weighted network *G* =< *V*, *E*, *ω* >. The probability distribution vector *Π* =< *π*_1_, …, *π*_(|*V*|)_ > is defined on set of all nodes of the network as follows:
  $$\begin{array}{c} \pi_{i}=\frac{\omega (g_{i})}{\sum_{g_{i}\in V}\omega (g_{i})}. \end{array}$$ (6)
  Then, the entropy of weighted graph *G* is calculated as follows:
  $$\begin{array}{c} En\left(G\right)=-\sum_{i=1}^{\left|V\right|}\pi_{i}\;\text{log}\;\pi_{i}. \end{array}$$ (7)
  We calculated the effect of each node *g*_*i*_ on network entropy as follows:
  $$\begin{array}{c} \varepsilon \left(g_{i}\right)=\left|En\left(G\right)-En\left(G\backslash g_{i}\right)\right|, \end{array}$$ (8)
  where *G*\*g*_*i*_ is the weighted network that is constructed with respect to the removal of node *g*_*i*_ and its connected edges from the network.

### 2.3 Machine learning method to select top mutated cancer genes

Since the problem of selecting the set of mutated candidate driver cancer genes is still an open question, it can be studied as a problem without an exact answer. Therefore, we utilized an effective unsupervised feature selection method to determine an efficient set of mutated cancer genes. Suppose that *X* = [*x*_*ij*_]_*m*×*n*_ represents the feature matrix and *x*_*ij*_ represents the *j*-th feature of the *i*-th sample (genes). We assigned a feature vector $\vec{p_{i}}=<x_{i1},\ldots ,x_{in}>$ to each sample and defined the column matrix *F*_*j*_ = [*x*_1*j*_, …, *x*_*mj*_]^*T*^ for the *j*-th feature. To find an appreciated score for each feature, we used the Laplacian Score for Feature Selection (LSFS) as an unsupervised machine learning method as follows:

Suppose that *S* = [*s*_*ij*_]_*m*×*m*_ indicates the weighted matrix where $s_{ij}=e^{-\frac{|\vec{p_{i}}-\vec{p_{j}}{|}^{2}}{t}}$ if the euclidean distance between two feature vectors $\vec{p_{i}}$ and $\vec{p_{j}}$ is less than *δ*. Also, suppose that *D* = [*d*_*i*_]_*m*×*m*_ is the diagonal matrix where $d_{i}=\sum_{k=1}^{m}s_{ik}$ and *L* = *D* − *S* is the Laplacian matrix. The Laplacian Score for each feature, *j*, is calculated as follows:

$$\begin{array}{c} L_{j}=\frac{{\tilde{F_{j}}}^{T}L\tilde{F_{j}}}{{\tilde{F_{j}}}^{T}D\tilde{F_{j}}}, \end{array}$$ (9)

where *J* = [1, 1, ‥, 1]^*T*^ and $\tilde{F_{j}}=F_{j}-\frac{{F_{j}}^{T}DJ}{J^{T}DJ}J$.

Finally, we calculated the LS for each mutated cancer gene *g*_*i*_ as follows:

$$\begin{array}{c} LS\left(g_{i}\right)=\sum_{j=1}^{n}x_{ij}L_{j}. \end{array}$$ (10)

The algorithm to calculate Laplacian Score (LS) for each mutated cancer gene is described in Algorithm 1. In this algorithm, we consider that *δ* = 5 and *t* = 100 respectively.

**Algorithm 1** The Laplacian Score for Feature Selection (LSFS)

**Require**: : Feature matrix *X* = [*x*_*ij*_]_*m*×*n*_.

1: Let $\vec{p_{i}}=<x_{i1},\ldots ,x_{in}>$ for each sample *i*

2: Let *F*_*j*_ = [*x*_1*j*_, …, *x*_*mj*_]^*T*^ for each feature *j*

3: **for** *i* ⇐ 1 to *m* **do**

4:  **for** *j* ⇐ 1 to *m* **do**

5:   **if** $|\vec{p_{i}}-\vec{p_{j}}|<\delta$ **then**

6:    $s_{ij}=e^{-\frac{|\vec{p_{i}}-\vec{p_{j}}{|}^{2}}{t}}$

7:   **else**

8:    *s*_*ij*_ = 0

9:   **end if**

10:  **end for**

11: **end for**

12: *S* = [*s*_*ij*_]_*m*×*m*_

13: *D* = [*d*_*i*_]_*m*×*m*_, where $d_{i}=\sum_{k=1}^{m}s_{ik}$

14: *L* = *D* − *S*

15: *J* = [1, 1, ‥, 1]^*T*^

16: **for** *j* ⇐ 1 to *n* **do**

17:  $\tilde{F_{j}}=F_{j}-\frac{{F_{j}}^{T}DJ}{J^{T}DJ}J$.

18:  $L_{j}=\frac{{\tilde{F_{j}}}^{T}L\tilde{F_{j}}}{{\tilde{F_{j}}}^{T}D\tilde{F_{j}}}$

19: **end for**

20: **for** *i* ⇐ 1 to *m* **do**

21:  $LS\left(g_{i}\right)=\sum_{j=1}^{n}x_{ij}L_{j}$

22: **end for**

### 2.4 Heuristic algorithm to identify specific modules for each cancer type

A biological network is constructed as an undirect weighted graph G=<V,E,W> where the set of nodes $V=\{g_{1},\ldots ,g_{N}\}$ is the *N* top mutated cancer genes regarding maximum LS values. Two mutated cancer genes *g*_*i*_ and *g*_*j*_ are connected through an edge *e*_*ij*_ if they participate in the same biological process or if there is physical interaction between them. The $W(g_{i})$ represents the weight of the mutated cancer gene with respect LS value. In this work, we define a cancer driver module as a dense sub-network in the biological graph with the maximum average LS of the nodes of the sub-graph. Since this problem is NP, we presented a heuristic algorithm *MG* to cluster the weighted network G. Suppose that S⊆V is the subset of nodes in the network. The neighborhood of S is defined as follows:

$$\begin{array}{c} C\left(S\right)=\left\{g_{i}\in V-S|\exists g_{j}\in S;g_{i}g_{j}\in E\right\} \end{array}$$

The *MG* algorithm first selects a node as a module. Then, the new module expands by adding a new node to the module regarding the average LS value of the nodes in this module and the LS values of adjacent nodes in the module. The *MG* algorithm adds a new adjacent node to a module such that the node’s weight is greater than the average weight of the module nodes. If the weight of all adjacent module nodes is less than the average module weight, the *MG* algorithm adds an adjacent node with the highest weight to the module with a small probability. The likelihood of reaching nodes with smaller weights decreases as the number of nodes in the module increases. The *MG* constructs a new module by selecting a new seed from the network nodes that have not been placed in a module and then expanding this node to get a new module. The *MG* algorithm extends modules to weighted graphs described in Algorithm 2. In this algorithm, we consider that *T* = 10 and *T*_*low*_ = 0.01 respectively.

**Algorithm 2** *MG* algorithm

**Require**: : The weighted graph G=<V,E,W>

**Require**: : The module S

1: **while** *T* < *T*_*low*_ **do**

2:  Select $g_{i}\in C\left(S\right)$

3:  Select *x* = *random*(0, 1)

4:  **if** $W\left(S\right)<W\left(g_{i}\right)$ **then**

5:   $S=S\cup g_{i}$

6:  **elseIf** $x<{\text{exp}}^{\frac{(W\left(g_{i}\right)-W\left(S\right)}{T}}$

7:   $S=S\cup g_{i}$

8:  **end if**

9:  *T* = 0.9 * *T*

10: **end while**

## 3 Results

### 3.1 Evaluation of LSFS algorithm and candidate mutated genes

In this study, we modeled different topological features for mutation networks with the help of the feature selection method. The output of our proposed LSFS algorithm shows us the significance of each feature. With the help of the LSFS algorithm, we selected the top six features with the significantly highest Laplacian Score. The LSFS algorithm’s result indicates that this work’s pre-defined topological features are significant and contain meaningful information about the network. Table 1 shows the Laplacian Score for each feature, respectively.

**Table 1:** The Laplace Score value for each feature. Our proposed model includes six topological features of the mutation network, which is made up of mutation frequency information on 15 types of cancer.

Topological Feature	Laplacian Score
Weight	0.452
Closeness	0.351
Betweenness	0.992
PageRank	0.32
Eigenvector	0.04
Entropy	0.05

#### 3.1.1 Evaluation of simulated data based on LS

In this study, we derived the simulated datasets using the prostate cancer-related genes introduced in TCGA [6]. In the first step, we collected prostate cancer-related genes, including growth factor pathway genes such as PTEN, P27, and NKX3.1, which increase cancer cell proliferation and include oncogenes such as AR. To create the simulated dataset related to prostate cancer, we selected 20 genes related to prostate cancer and 80 unrelated genes as samples. For these 100 genes, we defined 200 cases. We assigned prostate cancer-related genes with a rate of 40% and unrelated genes with a rate of 20% to cases. In this way, we constructed the simulated dataset. Then we used Algorithm 1 and assigned each sample (gene) score. The analysis in this part showed that the set of 20 high-score genes contains 12 genes related to prostate cancer (mTOR, PTEN, P27, NKX3.1, TP53, EP300, AR, KRAS, PIK3CA, KMT2D, APC, ARID1A). It shows that our algorithm works as well on simulated data as on real data.

#### 3.1.2 Evaluation of high score selected genes based on LS

**Fig 2:**
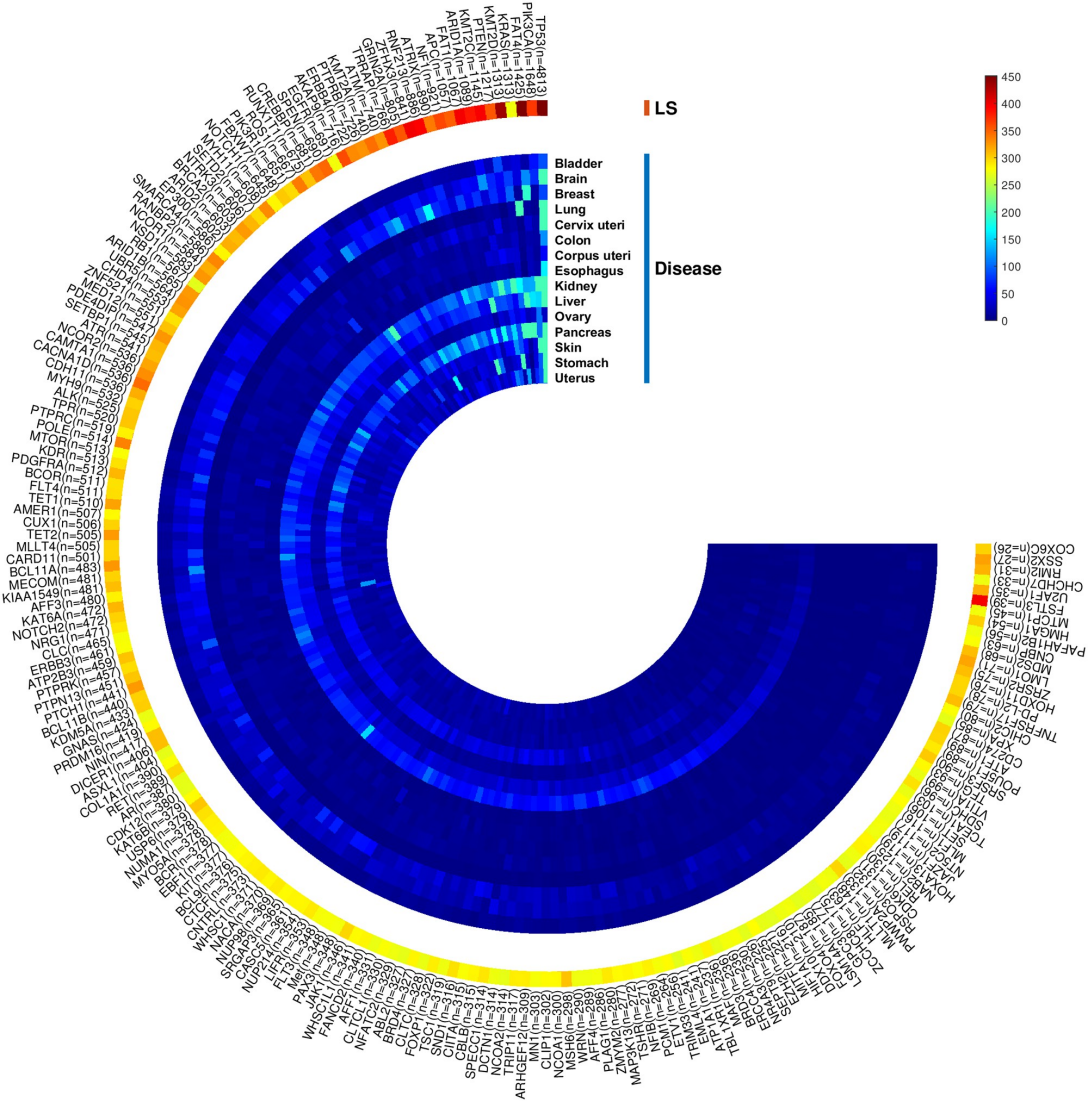
The LS value for 200 high-score genes is based on the number of their mutations in 15 different types of cancer, which are sorted counter-clockwise. This Fig shows the number of infrequently mutated genes with high LS as a result of our method with red color.

One of the significant challenges for existing methods is that they need extensive filtering of mutation data, which is limited to the most significantly mutated genes and focuses on predefined modules. Therefore, the mutual exclusivity signal can be biased toward recognizing gene sets where most of the coverage comes from highly mutated genes. Finding a new set of genes with essential properties, even if they have moderately or infrequently mutated, leads us to some new informative modules. To evaluate the LSFS algorithm’s performance, we apply LSFS to a representative set of mutations gathered from TCGA [6]. The proposed unsupervised machine learning method calculated the LS for each candidate mutated cancer gene in this set. Among 576 candidate mutated cancer genes, 200 genes with a higher LS than average LS were selected as high-score mutated cancer genes. Fig 2 shows the heat map of the number of mutations for each gene in each cancer and the value of the associated LS for these high-score genes. In Fig 2, we sorted 200 high-score genes based on the number of their mutations in 15 different types of cancer. Genes with high LS are highlighted in Fig 2. This Fig contains some of the frequently mutated genes such as TP53, FAT4, and KMT2C, and some of the infrequently mutated genes such as FSTL3, SSX2, and MDS2. We defined some genes with a frequency of less than 100 as infrequently mutated genes. Among 200 high-score genes, 25 genes were infrequently mutated genes, and among these 25 infrequently mutated genes, 18 genes had a high LS. Since most recent studies have focused on frequently mutated genes, we also studied the 18 infrequently mutated genes with high LS in addition to the frequently mutated genes. In the following, we present a list of these infrequently mutated genes with high LS.

- Follistatin-like 3 (FSTL3) is expressed in normal human tissues. Increasing evidence demonstrates that FSTL3 plays an essential role in regulating embryonic evolution, osteogenesis, glucose, and lipid metabolism. Furthermore, FSTL3 was found abundantly expressed in cell lung cancer and breast cancer and participates in tumor progression, containing invasion and metastasis. FSTL3 is an independent risk factor connected with the prognosis for different cancers [39].
- Recent studies demonstrate that Cytochrome c oxidase subunit 6c (COX6C) has a particular association with breast cancer, esophageal cancer, thyroid tumors, prostate cancer, uterine cancer, and melanoma. Several reports show that the differential expression of COX6C is associated with predicting some tumors and is expected to become one of the diagnostic markers of typical tumors [40].
- Recent studies demonstrated that SSX2 induces aging in different cells, as specified by classical aging features, including enlargement of the cytoplasm, cell growth arrest, and DNA double-strand breaks. SSX proteins are expressed in multiple types of tumors, such as 40% of melanomas and up to 65% of breast cancers. The SSX family comprises nine similar members, most likely redundant in their cellular functions [41].
- LMO1 belongs to the family of LIM-only domain genes (LMOs). Some studies have shown that LMO1 plays an essential role in the tumorigenesis of several types of cancer, including leukemia, breast cancer, and neuroblastoma. The author of [42] found that LMO1 was significantly over-expressed in non-small cell lung cancer (NSCLC) samples relative to normal adjacent tissue and that over-expression of LMO1 in NSCLC cells elevated cell proliferation, supporting an oncogenic function in NSCLC.
- The TNF receptor superfamily member 17 (TNFRSF17) is a gene that encodes a protein involved in B cell development and autoimmune response. This protein also plays a role in activating NF-*κ*B and MAPK8/JNK. Multiple types of mutations in TNFRSF17 have been shown in endometrial cancer, intestinal cancer, and skin cancer. On average, TNFRSF17 mutations are found in 0.50% of all cancers; the most common types are colorectal, colon cancer, glioblastoma, lung cancer, and malignant cancer melanomas [43].
- The Programmed cell death 1 ligand 2 (PD-L2) is a gene that encodes a protein that involves in the signal that is required for IFNG production and T-cell proliferation. Multiple types of mutations in PD-L2 have been observed in intestinal cancer, skin cancer, and stomach cancer [44]. On average, PD-L2 mutations are found in 0.83% of all cancers; the most common types are lung cancer, breast invasive ductal carcinoma, colon cancer, urothelial bladder carcinoma, and high-grade ovarian cancer [45].
- POU5F1 is associated with the pluripotency and proliferative potential of ESCs and germ cells. Previous studies have shown that POU5F1 plays a critical role in maintaining the normal stem cell self-renewal process. Several studies have noted the expression of POUF1 in human cancer cells such as breast cancer, ovarian cancer, and melanoma. Moreover, recent studies revealed that POU5F1 expression was significantly elevated in tumor tissues compared to non-cancerous tissues [46].
- The high mobility group A1 (HMGA1) gene has an essential role in embryonic development. Multiple studies have shown elevated HMGA1 expression in malignant cancer such as breast cancer, lung cancer, colorectal cancer, and uterine cancer. Collectively, these studies reveal that HMGA1 has an essential role in tumorigenesis and tumor progression [47].
- The Programmed death-ligand 1 (PD-L1), also known as CD274 on cancer cells, contributes to cancer immune escape. The PD-1/PD-L1 axis is the major speed-limiting step of the anti-cancer immune response for multiple cancer types. On average, CD274 mutations are found in 0.96% of all cancers; the most common types are breast cancer, gastric cancer, lung cancer, colon cancer, bladder cancer, and prostate cancer [48].
- All cancers have genome instability as a hallmark. RMI2 is an important element of the BLM-TopoIIIa-RMI1-RMI2 complex that supports genome stability. Several studies have shown the upregulated expression of RMI2, which is caused tumor progression in cervical cancer, lung cancer, and prostate cancer [49].
- The MDS2 is a gene that encodes a protein that functions in the onset of myelodysplastic syndrome (MDS). Multiple mutations in MDS2 have been shown in breast and ovarian cancer. On average, MDS2 mutations are found in 0.09% of all cancers; the most common types are breast cancer, appendix cancer, lung cancer, and colon cancer [50].
- Tropomyosin-receptor kinase fused (TFG) encodes a protein which is a maintained regulator of protein secretion that controls the export of materials from the endoplasmic reticulum. TFG belongs to the systems that control cell size and is implicated in apoptosis and cell proliferation regulatory mechanisms. The TFG fusion proteins play a role in oncogenesis, with the activity of TFG fusion proteins promoting tumor development. Multiple mutations in TFG have been shown in intestinal cancer, lung cancer, and stomach cancer. On average, TFG mutations are found in 0.19% of all cancers; the most common types are breast cancer, colon cancer, and lung cancer [51].
- The U2AF1 encodes for a member of the spliceosome. This protein plays a vital role in RNA splicing. Multiple mutations in U2AF1 can cause irregular expression patterns of some genes affected in cancer pathogenesis. On average, U2AF1 mutations are found in 1.5% of all cancers; the most common types are acute myeloid leukemia, colon cancer, and lung cancer [45].
- The SRSF3 is a member Ser/Arg-rich (SR) proteins family. As a potential diagnostic and prognostic biomarker, SRSF3 is overexpressed in various types of cancer, including cancer of the breast, retinoblastoma, ovarian cancer, gastric cancer, head and neck cell squamous, colorectal cancer, cervical cancer and hepatocellular carcinoma (HCC). Recent studies also show SRSF3 upregulation in mesenchymal tumors [52].
- Previous studies showed that ATF1 plays a crucial role in carcinogenesis and participates in multiple cellular processes, including cell transformation, cell cycle, DNA damage, and apoptosis. ATF1 is overexpressed in various types of cancer, including lymphomas, nasopharyngeal carcinoma, and melanoma. However, other studies have shown that ATF1 acts as a tumor suppressor in breast and colorectal cancer [53].
- SDHC is a gene that encodes a protein as a part of succinate dehydrogenase. Multiple types of mutations in SDHC have been observed in ovarian cancer and pancreatic cancer. On average, SDHC mutations are found in 1.5% of all cancers; the most common types are lung cancer, breast cancer, pancreatic cancer, colon cancer, and bladder cancer [45].
- HOXD11 is a member of HOX family, which encodes transcription factors that control different physiological processes. Recent studies have shown that HOXD11 is involved in tumor development and helps control gene expression. Multiple types of mutations and changes in expression in HOXD11 have been observed in lung cancer, Oral Squamous Cell Carcinoma, prostate cancer, ovarian cancer, and Head and Neck Squamous Cell Carcinoma. HOXD11 may also change cell growth, clonality, and metastatic potential in Ewing sarcoma [54].
- The protein coded by the ZRSR2 gene plays a vital role in RNA splicing. Multiple types of mutations in ZRSR2 have been observed in chronic myelomonocytic leukemia and chronic lymphocytic leukemia. These mutations can drive abnormal expression patterns of some genes involved in cancer pathogenesis. On average, ZRSR2 mutations are found in 1.2% of all cancers; the most common types are lung cancer, breast cancer, colon cancer, and ovarian cancer [45].

#### 3.1.3 Signaling pathways associated with high score genes

One of the effective strategies for finding appropriate therapeutic approaches for cancer is identifying molecular pathways and specifying important genes in these pathways. Finding a new set of infrequently mutated genes with important properties can identify new pathways in different cancers and introduce them for further study. Therefore, we looked into the signaling pathways related to these genes and presented more information about them in Table 2. We also studied the significant signaling pathways associated with our 200 top-selected mutated cancer-related genes. Table 3 shows some of the significant signaling pathways for these 200 top selected genes, the average LS of the genes for each of these pathways, and the *p* − *value* of them with the help of the Database for Annotation, Visualization, and Integrated Discovery (DAVID) [55]. In the following, we describe the association between these significant pathways and different types of cancer and the role of the driver gene in each pathway.

1. hsa04068: FoxO signaling pathway
  The first pathway with the highest score in Table 3 is the FoxO pathway. FoxO, as a family of transcription factors (FoxOs), has a direct role in cellular proliferation, oxidative stress response, and tumorigenesis. FoxOs are commonly inactivated by phosphorylation by several protein kinases such as AKT and PKB. The PI3K-Akt-FoxO signaling pathway has a significant role in various physiological processes such as cellular energy storage, growth, and survival [59]. One of the critical genes in this pathway that our algorithm has identified as a top gene is the transcriptional repressor factor CTCF. Recent studies show the effect of the CTCF factor on some cancers like prostate cancer by regulating the FoxO pathway [60]. The CTCF downregulates, or inhibition also governs the FoxO signal pathway and delays tumor growth. Therefore, the overexpression or genetic modification of CTCF affects the regulation of the FoxO pathway.
2. hsa04015: MAPK signaling pathway
  The second pathway is Mitogen-Activated Protein Kinase (MAPK). The cascade of this pathway is a highly protected module that plays an essential role in different processes such as cell proliferation, differentiation, and migration, and any deviation from the precise control of this signaling pathway initiates many diseases [61], including various types of cancer. This signaling pathway has different signaling paths to the cell nucleus that the protein members of the MAPK /ERK chain (or Ras-Raf-MEK-ERK) are recognized by our algorithm. Studies show that the ERK signaling pathway plays a crucial role in tumorigenesis, migration, and invasion [62].
3. hsa04151: PI3K-Akt signaling pathway
  The phosphatidylinositol 3-kinase-Protein Kinase-B (PI3K-AKT) plays an important role in intracellular physiological regulation. Various oncogenes and growth factor receptors stimulate this signaling pathway, such as MET, KIT, EGFR, and ERBB3, which our algorithm recognizes. This signaling pathway also contains important genes such as PI3K, PTEN, mTOR, and JAK, which our algorithm recognizes. These gens induce cell proliferation, stem cell differentiation, and tumor suppressors in metabolic regulation. Disruption of this pathway and mutations in any of these genes can exhaust the cell of the natural process. This pathway is involved in cancer progression, and dysregulation of the PI3K pathway can be crucial in the cancer process [63].
4. hsa04014: Ras signaling pathway
  One of the critical signaling pathways in cellular activity is the Ras signaling pathway. Abnormal activation of Ras proteins (including RRAS2, MRas, HRas, KRas, and NRas) is the primary stimulus of oncogenes that has an essential role in the main signaling pathway in cancer. Mutations of Ras proteins such as KRas, which our method recognizes, cause cancer development. Meantime, the mutation in the regulatory ligands like EGFR and EGR, as other top mutated genes identified by our algorithm, cause the activation of their downstream signaling cascade [64].
5. hsa04012: ERBB signaling pathway
  The ERBB tyrosine kinase family members demonstrate some of the most generally changed proteins in cancer. Anomalous tyrosine kinase activation via gene alterations can cause tumorigenesis, tumor growth, and progression. This signaling pathway also contains important genes such as PI3K, CBLB, mTOR, and KRAS, which our algorithm recognizes. Oncogenic alterations of genes encoding members of the ERBB family, leading to unusual ERBB signaling and driving tumor growth, have been reported in different types of cancer, such as breast, lung, and gastrointestinal cancers. Recent studies show that the ERBB family’s signaling abnormalities and mutations are essential in escaping antitumor immunity in the cell process [65].
6. hsa04072: mTOR signaling pathway
  Mammalian target of rapamycin (mTOR) participates in multiple signaling pathways and controls cell proliferation, autophagy, and apoptosis. Studies show that the mTOR signaling pathway is related to different diseases, such as various types of cancer. This signaling pathway is often activated in tumors and plays an essential role in tumor metabolism. Therefore, the mTOR signaling pathway could effectively target through anti-tumor therapy studies [66].

**Table 2:** Signaling pathways related to infrequently mutated genes.

Gene name	Signaling pathway
FSTL3	Ample FSTL3 expression promotes epithelial-mesenchymal transition (EMT) and improves aerobic glycolysis to positively affect cancer cells’ invasive and metastatic capacity by activating the *β*-Catenin pathway. Results of [39] show that FSTL3 could be a bridging molecule in the crosstalk between HIPPO/YAP1 and Wnt/*β*-Catenin pathways and that FSTL3 is an essential regulatory factor of the *β*-Catenin molecular mechanisms in cancer. [39].
COX6C	The expression level of COX6C was remarkably up-regulated in different cancers such as gastric and lung. It has been reported that overexpression of COX6C could promote the proliferation and decrease the apoptosis of cancer cells through activation of the oxidative phosphorylation pathway [40].
SSX2	It has been shown that the SSX proteins are activated in several critical mitogenic pathways, such as MAPK and Wnt [41].
LMO1	Studies show that LMO1 promoted the proliferation, aggression and migration of cancer cells by activation of NF-*κ*B pathway [56].
TNFRSF17	Recent studies show that overexpression of TNFRSF17 in cells activates the MAPKs pathway, specifically JNK and p38 kinase, NF-*κ*B, and Elk-1 [43].
PD-L2	Studies show the potential role of PD-L2 in regulating some pathways involved in cancer cell aggressiveness. They showed the modulation of ERK and Akt/PKB pathways are considered through PD-L2 [44].
POU5F1	Results of [46] demonstrate that IGF-IR/IRS-1/PI3K/AKT/GSK3*β* cascade-mediated regulation of POU5F1 and construction of *β*-catenin/POU5F1/SOX2 complex is essential for the retention of the self-renewal and tumorigenicity in cancer [46].
HMGA1	Recent studies show that HMGA1 s a critical transcription factor involved in multiple biological pathways, such as the TNF-*α*/NF-*κ*B, EGFR, Hippo, Ras/ERK, Akt, Wnt/beta-catenin and PI3-K/Akt pathways. In all of these pathways, HMGA1 targets various downstream genes [47].
PD-L1	PD-1/PD-L1 pathway regulates the induction and maintenance of immune tolerance within the tumor microenvironment. Recent studies show that PD-L1 is involved in multiple essential pathways, such as PI3K/AKT, MAPK, JAK/STAT, WNT, and NF-*κ*B pathways [48].
RMI2	Results of KEGG enrichment analysis indicated that RMI2 was significantly associated with the p53 signaling pathway [49].
MDS2	Recent studies show that knockdown of SPAG6 significantly increased the apoptosis of MDS cells by inducing the activation of tumor suppressor genes, such as p53 and PTEN. SPAG6 knockdown induced autophagy via the AMPK/mTOR/ULK1 signaling pathway in MDS2 cells, and inhibiting autophagy decreased SPAG6 knockdown-mediated apoptosis [50].
TFG	Recent studies show that TFG is involved in the NF-*κ*B and MAPK pathways, and activation of MAPK pathway occurs in various cancers, and the NF-kB pathway is essential in inhibition of apoptosis and treatment resistance in cancers [51].
U2AF1	The KEGG pathway enrichment analysis results showed that U2AF1 was involved in several biological pathways, such as FoxO and PI3K/Akt signaling pathways [51].
SRSF3	Recent studies show that SRSF3 as an oncogene manipulates various cell functions by regulating many pathways, such as p53, JNK, Ras, Wnt, and HER2 signaling pathways [52].
ATF1	Recent studies showed that ATF1 activates a subset of genes related to apoptosis, Wnt, TGF-*β*, and MAPK pathways, and these consequences could increase the risk of various cancers [53].
SDHC	Recent studies showed that SDH activity has a significant role in regulating oncogenic signaling pathways, such as those associated with NF-*κ*B [57].
HOXD11	Recent studies showed that HOXD11 is involved in various cancer-related signaling pathways such as cell cycle, DNA replication, ECM receptor interaction, and focal adhesion [54].
ZRSR2	Recent studies showed that ZRSR2 is involved in various cancer-related signaling pathways, such as TLR signaling pathway [58].

**Table 3:** Significant signaling pathways with the high average LS.

Signaling pathway	Ave. LS	No. gene	p-value
hsa04068: FoxO signaling pathway	314.4	10	16 * E^−4^
hsa04010: MAPK signaling pathway	308.8	15	4.4 * E^−4^
hsa04151: PI3K-Akt signaling pathway	302.3	20	6.6 * E^−6^
hsa04014: Ras signaling pathway	299.7	16	7.4 * E^−6^
hsa04012: ERBB signaling pathway	295	10	11 * E^−6^
hsa04072: mTOR signaling pathway	290.8	9	47 * E^−3^

**Fig 3:**
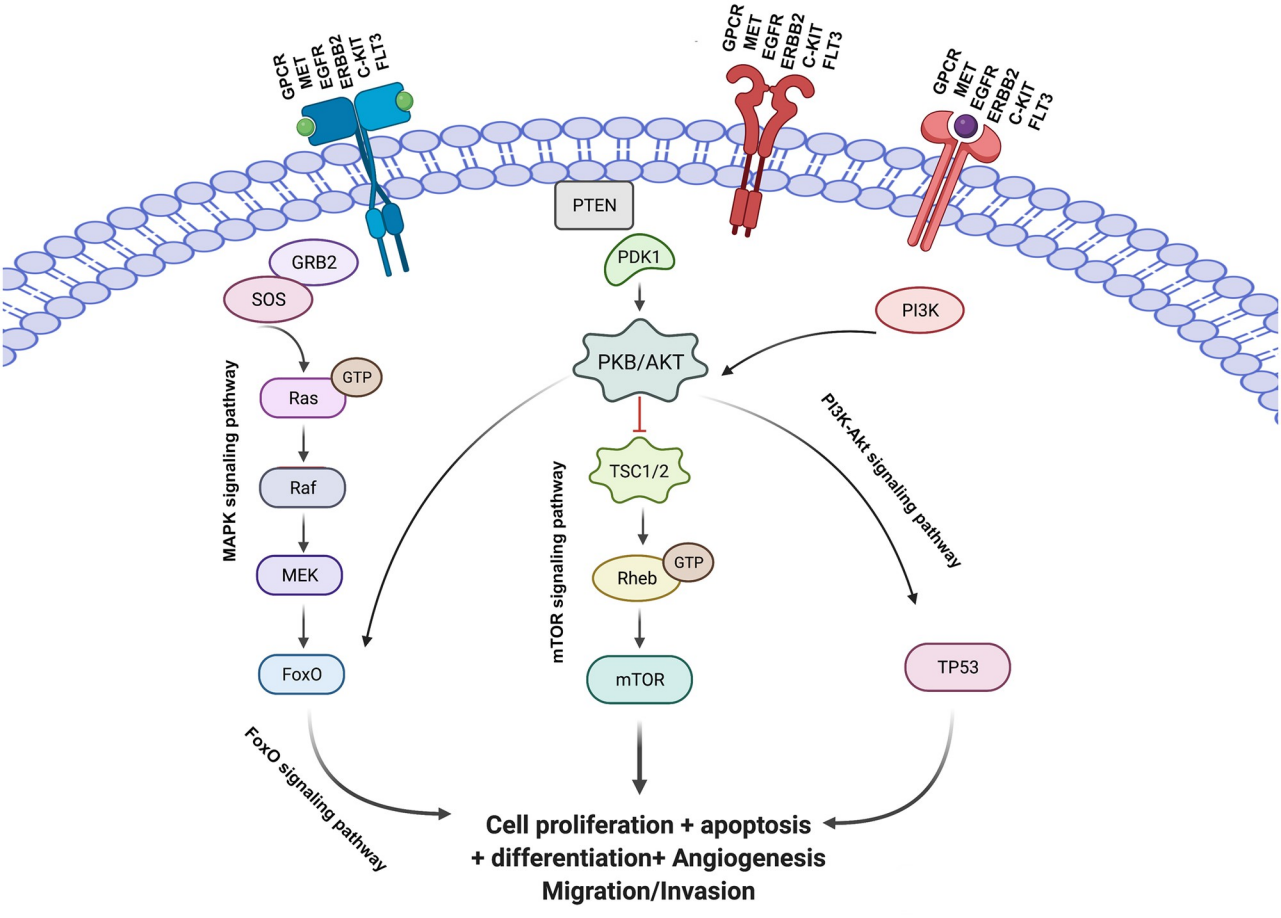
Cross-talk between significant signaling pathways for 200 top selected genes obtained by the LSFS algorithm and the role of these genes in the known cancer-related pathways.

Eight hallmark biological properties are essential for the emergence and survival of cancer: (1) replicate immortality, (2) sustained proliferation, (3) evasion of growth inhibitors, (4) invasion metastability, (5) death resistance, (6) angiogenesis, (7) immune evasion and (8) reprogrammed energy metabolism [45]. Three fundamental processes—cell survival, cell destiny, and genome maintenance—are supported by one or more of these core processes. One or more than one of the 12 critical signaling pathways provides support to these fundamental functions, and blocking these cancer-related pathways will disable cancer persistence and progression. MAPK, PI3K-Akt, Ras, mTOR are a number of these critical signaling pathways [45]. These 12 pathways’ components communicate with one another through a complex network of gene nodes, some of which are stimulatory and some of which are inhibitory. The importance of any of these nodes is dependent on that node’s degree, betweenness, closeness, and its neighbors as well. Systems analysis can optimize the disruption of a limited target route since many different genes can have limiting effects on a dominant signaling pathway. Additionally, many pathways likely need to be targeted due to the cross-talk across pathways and the cancer network’s resilience, which entails utilizing alternative or compensating secondary pathways. Fig 3 shows the cross-talk between all significant signaling pathways for these 200 top selected genes that are all among 12 critical cancer-related signaling pathways.

#### 3.1.4 Evaluation of LSFS accuracy

Comparing different driver gene identification methods is a major issue in driver gene identification, and it is challenging to show that one method is better than another. Since there is no specific and accurate benchmark to test the methods. In this work, to study the reality of the LSFS algorithm, we compared the driver genes obtained by LSFS with the driver genes of CGC, CGCpointMut, Rule, HCD, CTAT, and ShiBench as the benchmark for driver genes. Since only a part of the mutated genes introduced in section, 2.2 as set *V* = {*g*_1_, …, *g*_*n*_} included 576 genes, for the fairness of this comparison, we intersect each of these sets of genes with the set *V*. In total, 513 genes out of 576 in the CGC set, 112 genes out of 118 in the CGCpointMut, 158 genes out of 297 in the CTAT set, 148 genes out of 288 in the HCD set, 112 genes out of 124 genes in the Rule set, and 201 genes out of 232 genes in the ShiBench set is selected. To quantify this comparison, we used the following parameters. The number of genes that are correctly identified as the driver gene as True Positive (TP). The set of genes that are correctly identified as the non-driver (or passenger) gene as True Negative (TN). The genes that are incorrectly identified as the driver gene as False Positive (FP), and The genes that are incorrectly identified as non-driver gene False Negative (FN). The evaluation parameters of Precision (Pre), Recall (Re), and F-measure (F) are defined, respectively.

$$\begin{array}{c} Pre=\frac{TP}{TP+FP}. \end{array}$$ (11)

$$\begin{array}{c} Re=\frac{TP}{TP+FN}. \end{array}$$ (12)

$$\begin{array}{c} F=2*\frac{Pre*Re}{Pre+Re}. \end{array}$$ (13)

Table 4 shows an acceptable agreement between the LSFS algorithm and benchmark sets. Table 4 indicates that the LSFS algorithm with an F-measure value of 0.640 has the most agreement with the CGC set. After that, the ShiBench set, a filtered set of seven data sets and more reliable than the others, has the highest agreement.

**Table 4:** Statistical analysis for comparison of the LSFS algorithm with six benchmarks.

	TP	TN	FP	FN	Precision	Recall	F-meature
ShiBench-LSFS	77	251	123	124	0.385	0.383	0.384
CGC-LSFS	187	40	22	178	0.89	0.50	0.640
CGCpointMut-LSFS	42	305	158	70	0.21	0.375	0.269
CTAT-LSFS	59	276	141	99	0.29	0.373	0.329
HCD-LSFS	63	290	137	85	0.315	0.425	0.362
Rule-LSFS	43	306	157	69	0.215	0.383	0.275

**Fig 4:**
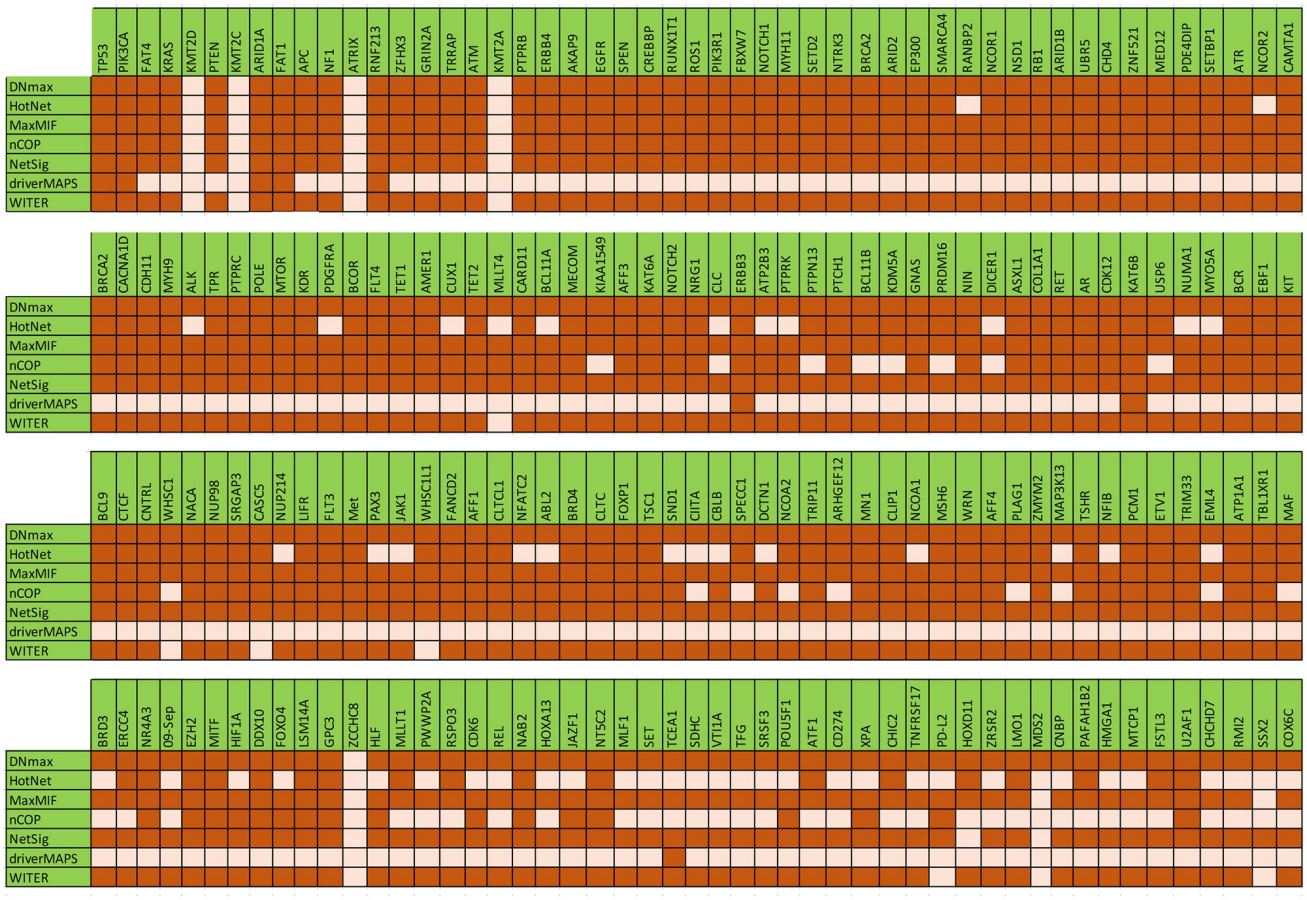
Comparison of the seven mentioned algorithms in the prediction of each of the 200 high-score genes as results of LSFS algorithm. The dark color shows the genes that are detected through each algorithm, and the light color demonstrates the genes that are not detected through these algorithms.

In order to have a better evaluation of the LSFS algorithm, we compared the LSFS algorithm with five algorithms: DNmax, HotNet, MaxMIF, nCOP, and NetSig, which are based on network evaluation, and the driverMAPS and WITER methods, which are based on the statistical method. In Fig 4, we have compared the LSFS algorithm with these seven algorithms. We looked into how well each of these algorithms predicted each of the 200 high-score genes that the LSFS algorithm identified as driver genes. In this Fig, each gene that is detected through each of the seven mentioned algorithms is denoted with a darker color, and genes that are not reported through these algorithms showed with a lighter color. Fig 4 shows that except for KMT2D, KMT2C, ATRX, KMT2A, ZCCHC8, MDS2, and SSX2 genes, the rest of the genes are recognized as driver genes for at least one of the cancer types by at least four other algorithms. We also investigated the role of mentioned seven genes in different cancers. A recent study [67] shows that the KMT2 family plays essential roles in controlling developmental pathways, and mutations in the genes encoding these proteins have been strongly associated with multiple cancers. Recent studies have supplied a more reasonable interpretation of the possible roles of disrupted KMT2 family proteins in cell growth abnormality and carcinogenesis. Authors in [68] showed that the tumor suppressor gene ATRX is frequently mutated in various tumors. ATRX is highly unresponsive to existing therapies. In [68], they performed a genome-wide synthetic lethal screen using CRISPR-Cas9 genome editing to specify potential therapeutic targets for ATRX-mutated cancers. Other studies [41, 50, 69] showed that driver gene mutations in multiple cancers, such as ZCCHC8, MDS2, and SSX2, were considered independent, mutually exclusive events. These studies offered that patients with mutations in these genes could benefit from targeted therapy for these genes.

**Fig 5:**
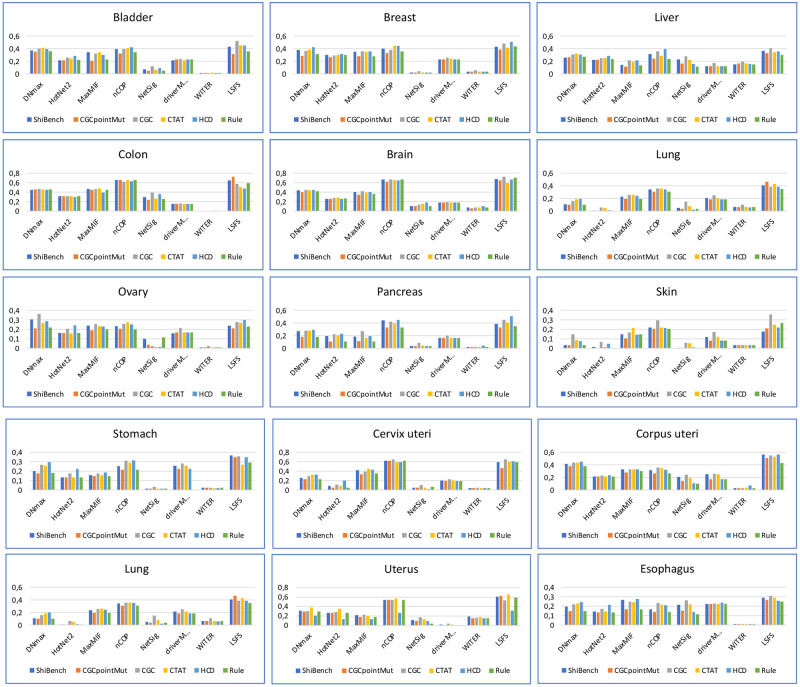
Comparison of the F-measure for each benchmark in LSFS algorithm and seven mentioned algorithms.

To better evaluate the LSFS algorithm, we have built 15 mutated networks related to 15 types of cancer and run the LSFS algorithm for these 15 types separately. Then, we compared the results of our algorithm and the other mentioned methods on each benchmark set. Fig 5 shows the value of the F-measure for each benchmark and each algorithm separately. As shown in Figs 4 and 5, WITER and NetSig algorithms could not show acceptable performance in any of the benchmarks. DNmax, HotNet2, MaxMIF, and driverMAPS algorithms have similar and proper performance based on F-measure. Fig 5 shows the significant superiority of the nCOP and LSFS algorithms based on the F-measure. However, the superiority of LSFS is apparent in most cancers and all benchmarks. Figs 4 and 5 show that the LSFS is superior to other algorithms and includes different and more complete results than other methods, even the nCOP.

### 3.2 Evaluation of MG algorithm and candidate driver modules

#### 3.2.1 Evaluation of MG algorithm result based on random sets

In the MG algorithm, we propose a method to find dense subgraphs with the highest average weight of nodes. Since finding sets with such characteristics is an NP problem, it is impossible to find an accurate and exact answer for this problem. Therefore, we have presented a heuristic algorithm to find solutions. To evaluate the result of the MG algorithm, we have compared the result of the MG algorithm with 1000 random subgraphs with the same size of seven modules. For each module of size *n*, we generate 1000 subgraphs of the set *C* = {*g*_1_, *g*_2_, …*g*_*N*_} (*N* is the number of top mutated genes) as samples. Suppose that *N*_*i*_ where *i* = 1, …, 1000 represents the average LS of the *i*-th sample and *M*_*i*_ where *i* = 1, …, 1000 represents the density of the *i*-th sample. We defined the density of a subgraph as the following:

$$\begin{array}{c} D\left(H\right)=\frac{2|E(H)|}{\left|V(H)|(|V(H)|-1\right)}. \end{array}$$ (14)

Which *H* =< *V*(*H*), *E*(*H*) > is a subgraph produced by each subset *V*(*H*) of the weighted graph G. Let’s assume that $\tilde{N}$ and $\tilde{M}$ are the average LS and module density, respectively. Now suppose

$$\begin{array}{c} X=\{i|N_{i}>\tilde{N}\;\&\;M_{i}>\tilde{M}\} \end{array}$$ (15)

It represents the random sets that perform better than our assumed module. The null hypothesis, *H*_0_, is the insignificant subgraphs of size *n*, and the alternative hypothesis, *H*_1_, is the selected subgraphs of size *n* that are significant. We define the Exceeding Value (*EV*) of each module as follows:

$$\begin{array}{c} EV=\frac{\left|X\right|}{1000}. \end{array}$$ (16)

that |*X*| represents the number of members of the set *X*. If *EV* < *α*, we reject the null hypothesis *H*_0_ (assuming the *α* threshold to be 0.05). The *EV* values for our eight selected modules are reported in Table 5. The values in Table 5 show that the selected sets represent significantly better than the random sets.

**Table 5:** The *EV* values for eight selected modules.

	Module 1	Module 2	Module 3	Module 4	Module 5	Module 6	Module 7	Module 8
|*X*|	1	1	1	1	2	1	1	1
EV	0.001	0.001	0.001	0.001	0.002	0.001	0.001	0.001

**Fig 6:**
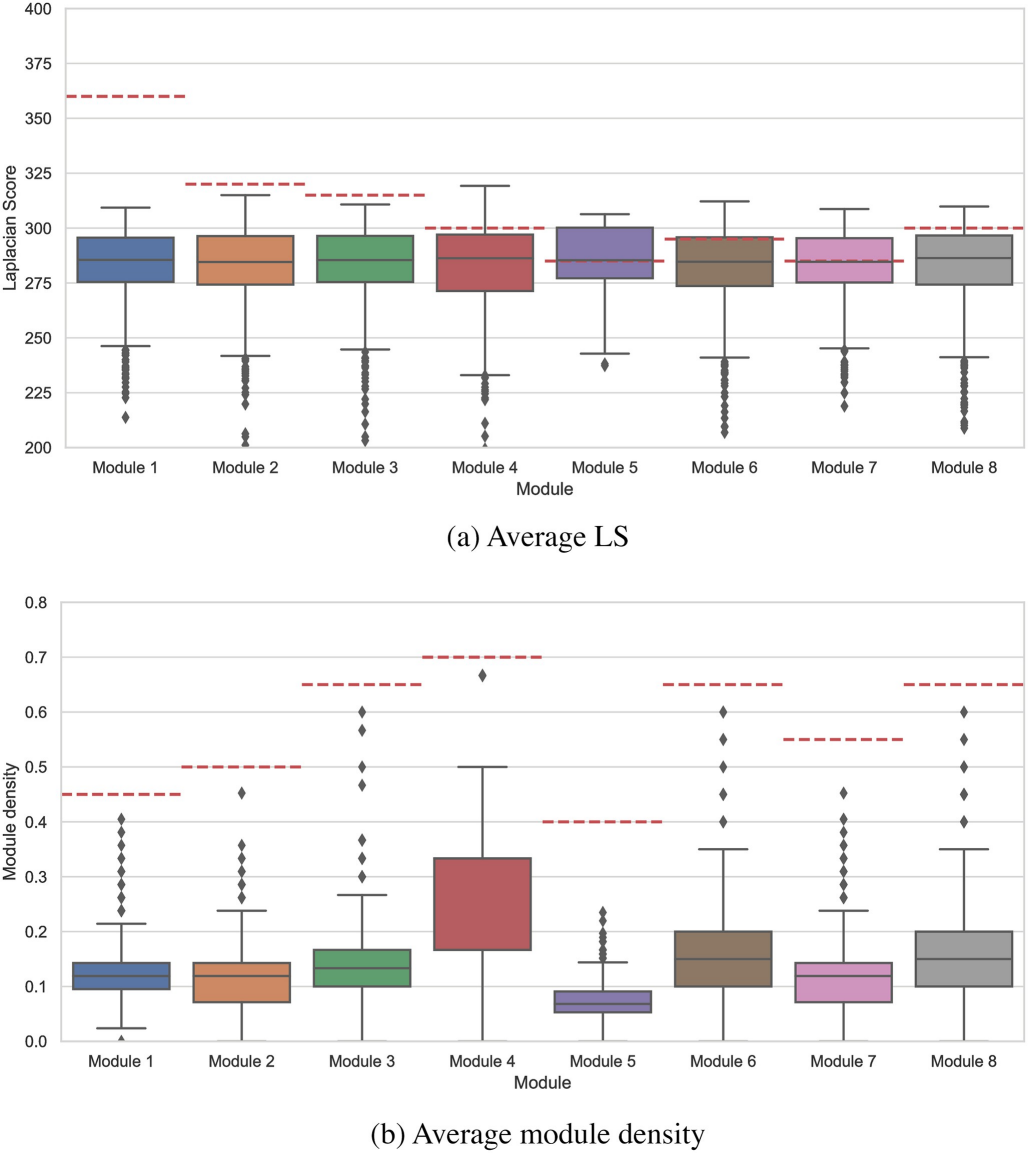
The boxplot diagram for each random set’s average LS (a) and average module density (b). The dashed line shows the corresponding value of LS and module density for each of our selected modules.

Fig 6(a) shows the boxplot diagram for each random set’s average LS and Fig 6(b) shows average module density. The dashed line in this Fig shows the corresponding value of LS and module density for each of our selected modules. Fig 6 shows that our selected modules have a high mean value of LS and significantly high density.

#### 3.2.2 Evaluation of the proposed driver modules based on Gene Ontology

**Fig 7:**
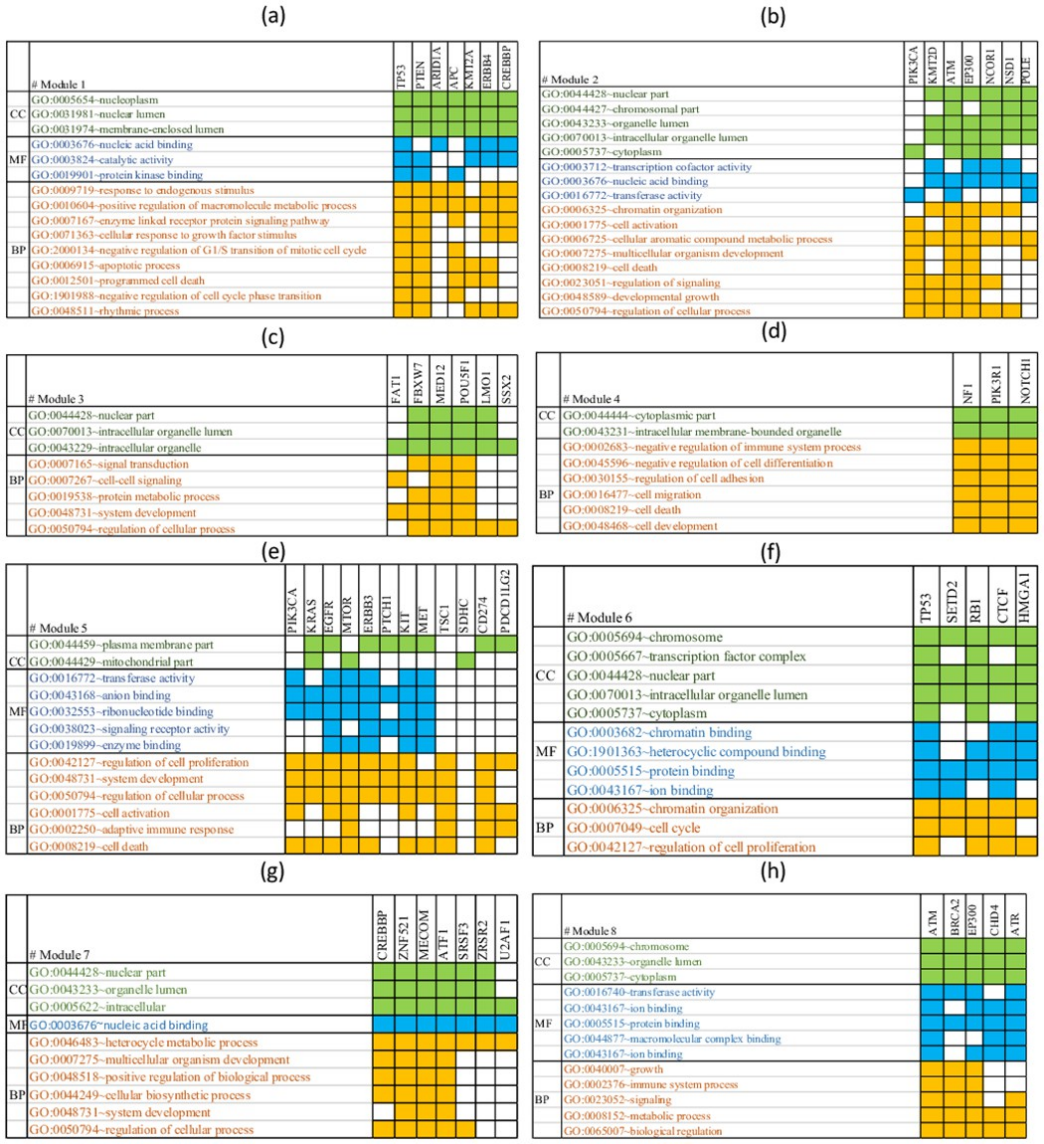
Significant GO terms for each module. CC, MF, and BP represent cellular components, molecular functions, and biological functions, respectively.

One of the best ways to justify candidate cancer driver modules is to evaluate their biological processes and functional modules. For this purpose, we have performed an analysis of the GO term annotations of the obtained driver modules from our method with the help DAVID tool [55]. Fig 7 shows significant GO terms for each driver module resulting from DAVID. In this Fig, CC, MF, and BP represent cellular components, molecular functions, and biological functions, respectively. Fig 7 shows that the genes in each module participate in multiple important CC, MF and BP related to cancer as well. In the following, we describe detailed information about each module and its biological properties.

The MG algorithm for the first driver module as the most important driver module selects the TP53 gene as a seed and expands this module by adding PTEN, ARID1A, APC, KMT2A, ERBB4, and CREBBP genes. The average LS of genes in this module is 360.58. The accumulation of these genes is higher in the nucleoplasm and nuclear lumen region. These genes also participate in various functions, including binding to nucleotide acid and catalytic activity [55]. From the above genes, genes such as ATP, PTEN, and TP53 have the function of binding to protein kinases. These genes are active in many biological processes, some mentioned in Fig 7(a), including GO:0006915 ∼ apoptotic process and GO:0012501 ∼ programmed cell death.

The MG algorithm for the second driver module selects the PIK3CA gene as a seed and expands this module by adding KMT2D, ATM, EP300, NCOR1, NSD1, and POLE genes. The average LS of genes in this module is 320.75. Most of these genes are concentrated in the intracellular organelle lumen or cytoplasm, and all of them have an activity of GO:0003676 ∼ nucleic acid-binding. These genes are involved in critical biological processes such as GO:0001775 ∼ cell activation, GO:0048589 developmental growth, and GO:0008219 ∼ cell death. Fig 7(b) shows significant GO terms associated with the genes of this module.

The MG algorithm for the third driver module selects the FAT1 gene as a seed and expands this module by adding FBXW7, MED12, POUF1, LMO1, and SSX2 genes. The average LS of genes in this module is 315.48. All genes in this module are known as regulators of biological processes and play a role in signal transmission. Fig 7(c) shows significant GO terms associated with the genes of this module.

The MG algorithm for the fourth module selects the NF1 gene as a seed and expands this module by adding PIK3R1 and NOTCH1 genes. The average LS of genes in this module is 301.88. The accumulation of these genes is cytoplasm and an intracellular membrane-bounded organelle. These genes are also involved in critical biological processes such as GO:0016477 ∼ cell migration, GO:0048468 ∼ cell development, and GO:0008219 ∼ cell death. Fig 7(d) shows significant GO terms associated with the genes of this module.

The MG algorithm for the fifth driver module selects the KRAS gene as a seed and expands this module by adding PIK3CA, EGFR, mTOR, ERBB3, PTCH1, KIT, MET, TSC1, CD274, PDCD1LG2 genes. The average LS of genes in this module is 286.72. These genes are involved in critical biological processes such as GO:0002250 ∼ adaptive immune response, GO:0008219 ∼ cell death, and GO:0042127 ∼ regulation of cell proliferation. Fig 7(e) shows significant GO terms associated with the genes of this module.

The MG algorithm for the sixth driver module selects the SETD2 gene as a seed and expands this module by adding TP53, RB1, CTCF, and HMGA1 genes. The average LS of genes in this module is 292.53. These genes are involved in critical biological processes such as GO:0042127 ∼ regulation of cell proliferation and GO:0007049 ∼ cell cycle. Fig 7(f) shows significant GO terms associated with the genes of this module.

The MG algorithm for the seventh driver module selects the CREBBP gene as a seed and expands this module by adding ZNF521, MECOM, ATF1, SRSF3, ZRSR2, and U2AF1 genes. The average LS of genes in this module is 284.07. In this cluster, some genes, such as ATF1, SRSF3, and ZRSR2, have fewer mutations than other genes. These genes are involved in critical biological processes such as GO:0048518 ∼ positive regulation of biological process and GO:0048731∼ system development. Fig 7(g) shows significant GO terms associated with the genes of this module.

The MG algorithm for the eighth driver module selects the BRCA2 gene as a seed and expands this module by adding ATM, EP300, CHD4, and ATR genes. The average LS of genes in this module is 300.52. The accumulation of these genes is more in the chromosome and cytoplasm regions and they are involved in critical biological processes such as GO:0040007 ∼ growth and GO:0002376 ∼ immune system process. Fig 7(h) shows significant GO terms associated with the genes of this module.

Gene Set Enrichment Analysis (GSEA) is one of the most common approaches for evaluating driver modules. In this approach, Eq 17 is used to show the significance of the driver modules obtained based on the set of well-known cancer-related pathways.

p-value=(Kk)(N-Kn-k)(Nn). (17)

*N* is the total number of mutated genes in set *V*, which includes 576 mutated genes. *K* is the number of genes in an understudied well-known cancer-related pathway. *n* is the number of driver genes of the understudy module, and *k* is the number of genes in the driver module that are in an understudied well-known cancer-related pathway. We selected eleven cancer-related pathways for this evaluation. We have highlighted the *p* − *value* of less than 0.05 corresponding to each pathway in Table 6 for each driver module.

**Table 6:** GSEA for driver modules evaluation.

	Module 1	Module 2	Module 3	Module 4	Module 5	Module 6	Module 7	Module 8
FoxO signaling pathway	**0.04**	**0.004**	**0.0071**	0.14	**0.02**	0.75	0.27	**0.02**
Wnt signaling pathway	**0.002**	0.235	0.764	0.874	0.582	0.7834	0.235	0.183
MAPK signaling pathway	0.0856	0.562	0.61	0.2007	**0.0019**	0.66	**0.011**	**0.02**
p53 signaling pathway	**0.01**	0.19	0.81	0.9	0.66	**0.014**	0.78	**0.009**
Estrogen signaling pathway	0.7	0.23	0.76	0.12	**0.011**	0.79	0.73	0.779
Ras signaling pathway	0.6	0.3	0.67	**0.01**	**0.004**	0.72	0.64	0.72
ErbB signaling pathway	0.24	0.24	0.74	0.12	**0.0009**	0.78	0.71	0.78
mTOR signaling pathway	0.19	0.19	0.8	**0.09**	**0.0042**	0.8	0.77	0.83
Pathways in cancer	**0.03**	0.29	0.23	0.1	**0.02**	0.22	0.29	0.22
PI3K-Akt signaling pathway	**0.02**	0.44	**0.049**	0.262	**0.0069**	0.347	0.44	0.55
VEGF signaling pathway	0.84	0.14	0.86	0.7	**0.02**	0.88	0.84	0.88

**Fig 8:**
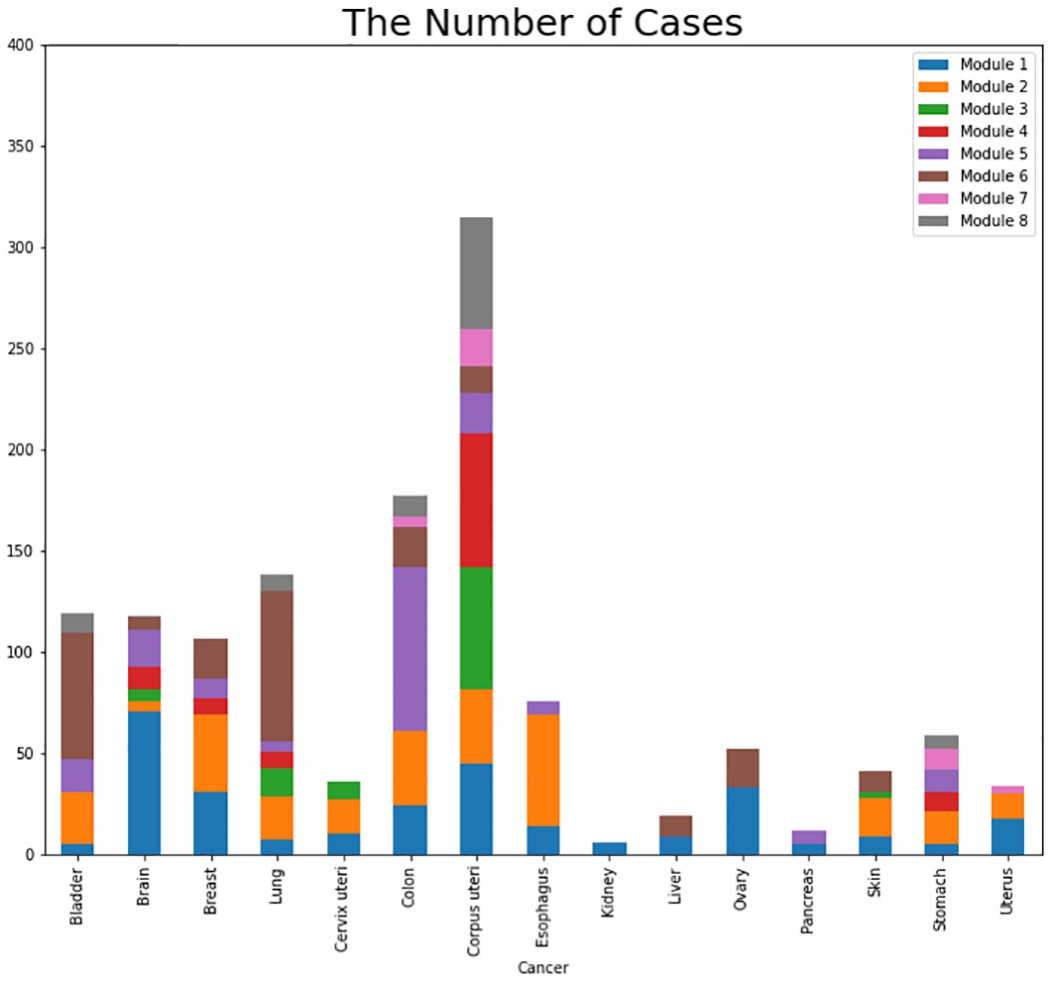
The number of cases in each module in each cancer separately. In each type of cancer, the number of cases with a simultaneous mutation in each module’s genes is shown in a distinct color.

We also statistically studied the genes in each module that simultaneously have several mutations in multiple cases. Table 7 shows the set of modulated genes for each cancer. The first column of Table 7 shows the module number. The second and the third columns show the genes found in more than 10% and less than 10% of the cases simultaneously. For example, among 1379 patients with breast cancer, 65 patients in module 2 had mutations in the NCOR1 gene. Of these 65 patients, 38 patients had mutations in NCOR1 and PIK3CA genes simultaneously. The important point to finding the module like previous studies, we have examined the number of simultaneous mutations in the most significant number of cases. Genes with fewer mutations are expected to participate in fewer modules. Therefore, if we want to see genes with fewer mutations in our modules, we should define other criteria than the number of mutations. Fig 8 shows the number of cases in the modules introduced by our algorithm in each cancer separately. For example, modules 3, 4, and 8 are found in more cases than the other five modules.

**Table 7:** A set of obtained important modules for each cancer separately.

Cancer type	Modules No.	Genes Mutated in > 10% cases	Genes Mutated in < 10% cases
Bladder	Module 1	TP53, PTEN	ARID1A
	Module 2	KMT2D, EP300	
	Module 5		PIK3CA, ERBB3
	Module 6	TP53, RB1	
	Module 8	EP300	ATM
Brain	Module 1	TP53	PTEN
	Module 2	KMT2D	POLE
	Module 3		FAT1, FBXW7
	Module 4	NF1, PIK3R1	
	Module 5	EGFR	PIK3CA
	Module 6	SETD2, RB1	HMGA1
Breast	Module 1	TP53, PTEN	ARID1A
	Module 2	PIK3CA	NCOR1
	Module 4		NF1, PIK3R1
	Module 5	PIK3CA	KRAS,
	Module 6	TP53	SETD2
Lung	Module 1	TP53, PTEN, ARID1A	
	Module 2	KMT2D, ATM,	
	Module 3	FAT1, FBXW7	
	Module 4	NF1, NOTCH1	
	Module 5	KRAS, EGFR	SDHC
	Module 6	TP53, RB1	
	Module 8	ATM	EP300
Cervix uteri	Module 1		PTEN, ARID1A
	Module 2		PIK3CA, EP300
	Module 3		FAT1, FBXW7
Colon	Module 1	TP53, PTEN	
	Module 2	POLE	KMT2D
	Module 5	KRAS	PIK3CA,
	Module 6	SETD2	TP53
	Module 7	CREBBP	ATF1
	Module 8	ATM, EP300,CHD4	
Corpus uteri	Module 1	PTEN, ARID1A, KMT2A	TP53, ERBB4
	Module 2	PIK3CA, KMT2D, NSD1, POLE	ATM
	Module 3	FAT1, FBXW7	
	Module 4	NF1, PIK3CA	
	Module 5	PIK3CA, mTOR, ERBB3, PTCH1	EGFR, KIT, MET, TSC1
	Module 6	SETD2, RB1, CTCF	TP53
	Module 7	CREBBP	ATF1
	Module 8	CHD4	ATM
Esophagus	Module 1		TP53, ARID1A
	Module 2	KMT2D, ATM	PIK3CA
	Module 5	KRAS	PIK3CA
Kidney	Module 1		TP53, PTEN
Liver	Module 1		TP53, ARID1A
	Module 6		TP53, SETD2
Ovary	Module 1	TP53, KMT2A	
	Module 6	TP53	SETD2
Pancreas	Module 1	TP53	ARID1A
	Module 5	KRAS	PIK3CA
Skin	Module 1		TP53, PTEN
	Module 2	KMT2D, ATM	
	Module 3	POUSF1, SSX2	
	Module 6		TP53, SETD2
Stomach	Module 1	TP53, PTEN	ARID1A, ERBB4
	Module 2		PIK3CA, KMT2D, ATM
	Module 4		NF1, PIK3R1
	Module 5		PIK3CA, KRAS, TSC1
	Module 7		CREBBP, ZNF512
	Module 8	ATM, CHD4, ART	
Uterus	Module 1		TP53, PTEN
	Module 2		PIK3CA, KMT2D
	Module 7	ZNF512	ZRSR2

## Conclusion and discussion

New sequencing technologies and improving genomics data help us identify cancer-related genes and modules in various cancers. Most previous studies focus on using statistical methods to identify high-frequency mutation genes. Finding these mutation genes is important in determining the cancer progression mechanism. The critical point is that some critical genes do not have high mutation frequencies and can not be identified depending on the number of mutations and statistical techniques. In this study, we used a machine learning method to find important cancer genes with low-frequency mutations along with the driver genes with high-frequency mutations. For this purpose, we extracted 576 cancer-related genes for 15 common cancers reported on TCGA and constructed a weighted graph for the corresponding mutations of these genes. The weight of the associated edge between two genes in this network is based on the number of common cases that contain these mutated genes simultaneously. Since the problem of finding candidate driver genes is still an open question, it can be studied as a problem without an exact answer. We used an unsupervised learning method to determine an efficient set of mutated cancer genes to find an appropriate response to this question. We defined six informative features for each gene and calculated the score for these features with the help of the mentioned unsupervised machine learning method for each gene. Afterward, we introduced 200 high-score genes with meaningful relationships to cancer as candidate genes for more investigation. We comprehensively compared our methods with seven different algorithms, simulated data, and multiple benchmarks. Our results showed that the proposed method has an outstanding performance. Our method proposed some genes, such as TP53, FAT4, and KMT2C, with high-frequency mutations as high-score genes that are presented through other statistical methods. In addition to these genes, our method also identified some genes, such as FSTL3, SSX2, and MDS2, with low-frequency mutations. We briefly studied these genes with low-frequency mutations and examined the association of each of these genes with different types of cancer. In addition, we studied the KEGG signaling pathways of the set of high-score genes. We also examined the roles of these high-score genes and the effects of mutations and abnormalities of these genes in the proposed set of signaling pathways in the different cellular processes such as proliferation and migration.

Genomic analysis of different types of mutation in genes indicates the mutation heterogeneity problem. Genes should be accepted as a module rather than as individuals in order to solve this heterogeneity issue. We used the knowledge of gene binding in protein-protein interaction networks and the information on the biological processes of each of these genes to detect the high-score genes and identify cancer-stimulating modules with high accuracy. For this purpose, we created a network based on information about the physical interactions of genes and the biological processes of these genes. We added weight to each node of this network with the help of the LS. Then, we proposed a heuristic algorithm and clustered the network. We introduced 8 top modules with the highest LS. To better understand the biological function of the genes in each module, we analyzed the GO term annotations of the genes for each module with the help of the DAVID tool. We have used GSEA as one of the most common approaches for evaluating driver modules and showed significant p-value corresponding to each pathway related to each proposed module.

It can be concluded that the methods that filtrate mutation data based on the most mutated genes and pre-defined network modules may lose important information about genes with a lower frequency of mutations. The mutual exclusivity signal may be biased toward recognizing gene sets that have a large proportion of their coverage in highly mutated genes. Although cancer-related genes have been shown to be involved in numerous pathways, few methods have been developed to identify the candidate driver gene sets with different mutation frequencies. We proposed a method to detect candidate driver genes with varying mutation frequencies and showed their critical role in cancer progression.
